# An Evaluation of the Anti-Inflammatory Effects of a Thai Traditional Polyherbal Recipe TPDM6315 in LPS-Induced RAW264.7 Macrophages and TNF-α-Induced 3T3-L1 Adipocytes

**DOI:** 10.3390/cimb45060311

**Published:** 2023-06-05

**Authors:** Phetpawi Subin, Pattraporn Sabuhom, Alisa Naladta, Prathan Luecha, Somsak Nualkaew, Natsajee Nualkaew

**Affiliations:** 1 Division of Pharmacognosy and Toxicology, Faculty of Pharmaceutical Sciences, Khon Kaen University, Khon Kaen 40002, Thailand; su_phetpawi@kkumail.com (P.S.); pattraporn.sb@kkumail.com (P.S.); prathanl@kku.ac.th (P.L.); 2 Department of Biochemistry, Faculty of Sciences, Khon Kaen University, Khon Kaen 40002, Thailand; alisa_n@kkumail.com; 3 Pharmaceutical Chemistry and Natural Product Research Unit, Faculty of Pharmacy, Mahasarakham University, Mahasarakham 44150, Thailand; somsak.n@msu.ac.th

**Keywords:** Thai traditional recipe, anti-inflammation, anti-obesity-induced inflammation, nitric oxide inhibition, adiponectin, PPAR-gamma

## Abstract

TPDM6315 is an antipyretic Thai herbal recipe that contains several herbs with anti-inflammatory and anti-obesity activities. This study aimed to investigate the anti-inflammatory effects of TPDM6315 extracts in lipopolysaccharide (LPS)-induced RAW264.7 macrophages and TNF-α-induced 3T3-L1 adipocytes, and the effects of TPDM6315 extracts on lipid accumulation in 3T3-L1 adipocytes. The results showed that the TPDM6315 extracts reduced the nitric oxide production and downregulated the iNOS, IL-6, PGE_2_, and TNF-α genes regulating fever in LPS-stimulated RAW264.7 macrophages. The treatment of 3T3-L1 pre-adipocytes with TPDM6315 extracts during a differentiation to the adipocytes resulted in the decreasing of the cellular lipid accumulation in adipocytes. The ethanolic extract (10 µg/mL) increased the mRNA level of adiponectin (the anti-inflammatory adipokine) and upregulated the PPAR-γ in the TNF-α induced adipocytes. These findings provide evidence-based support for the traditional use of TPDM6315 as an anti-pyretic for fever originating from inflammation. The anti-obesity and anti-inflammatory actions of TPDM6315 in TNF-α induced adipocytes suggest that this herbal recipe could be useful for the treatment of metabolic syndrome disorders caused by obesity. Further investigations into the modes of action of TPDM6315 are needed for developing health products to prevent or regulate disorders resulting from inflammation.

## 1. Introduction

Thai traditional medicines are usually composed of a mixture of several herbs with different functions. These include the main herbs that directly treat an illness, as well as the assistant herbs that enhance the actions of these main herbs, relieve symptoms, or restore the body balance of a patient. The well-documented Thai traditional recipes in the national Thai traditional textbook have been self-approved for their efficacy and safety based on their long-term use since ancient times. However, scientific evidence is required for more confidence in their use by health professionals and patients.

Fever results from cellular inflammation. Lipopolysaccharide (LPS) is a bacteria-derived exogenous pyrogen that triggers the production of tumor necrosis factor-alpha (TNF-α), stimulates cyclooxygenases to produce prostaglandin E_2_ (PGE_2_), and subsequently raises the body’s temperature. Furthermore, the gene expressions of the cytokines and pro-inflammatory mediators associated with fever, including inducible nitric oxide synthase (iNOS), TNF-α, PGE_2_, and interleukin-6 (IL-6), are upregulated [1].

Adipose tissue mainly consists of adipocytes that are differentiated from preadipocytes. The major adipogenic transcription factors involved in adipocyte differentiation and maintenance are peroxisome proliferator-activated receptor gamma (PPAR-γ) and CCAAT/enhancer-binding protein alpha (C/EBPα). PPAR-γ plays a crucial role in the terminal differentiation of adipocytes and helps to maintain their differentiated state. C/EBPα, on the other hand, acts in conjunction with PPAR to promote the formation of mature adipocytes [2]. Adipocytes also secrete adiponectin, which enhances insulin sensitivity.

The accumulation of excessive adipose tissue leads to obesity, which is, in turn, a critical risk factor for the development of type II diabetes and other metabolic diseases. Obesity is also associated with increased amounts of inflammatory mediators. Hence, anti-inflammatory agents are a potential alternative to obesity treatments [3]. There is also a correlation between the inflammation of adipocytes and insulin resistance. TNF-α, a pro-inflammatory cytokine, plays a critical role in insulin resistance due to obesity [4]. The elevation of TNF-α and IL-6 due to obesity causes a decrease in glucose transfer protein type 4 (GLUT4) and lower adiponectin levels [5].

TPDM6315 is a traditional Thai recipe that is in the traditional Thai medicine textbook and is used to regulate the body imbalance that appears as fever. Interestingly, 10 of the 15 herbs included in this recipe possess anti-inflammatory and/or anti-fever properties related to inflammation, and 9 of these 15 herbs have anti-obesity properties, as shown in Table 1. This indicates that the attenuating effects of this recipe on inflammation are likely to be behind its anti-pyretic properties. Furthermore, since obesity is associated with the inflammation of adipocytes, as well as the development of insulin resistance and type II diabetes mellitus [6], we determined the anti-inflammatory properties of TPDM6315 extracts against TNF-α induced adipocytes.

To date, there has been no investigation into the anti-inflammatory and anti-obesity effects of TPDM6315 based on its traditional use as an anti-pyretic. This work investigated TPDM6315 extracts for their anti-inflammatory properties in LPS-stimulated RAW264.7 macrophages and TNF-α-stimulated 3T3-L1 adipocytes, as well as the potential anti-obesity activities in normal 3T3-L1 adipocytes. Understanding these activities may support the traditional use of this formula and provide potential for the further development of health products to prevent or alleviate the consequences of metabolic syndrome diseases.

## 2. Materials and Methods

### 2.1. Chemicals

All the general reagents and solvents were of an analytical grade. Lipopolysaccharide (LPS), N (ω)-nitro-L-arginine methyl ester (L-NAME), NaNO_2_, dexamethasone, 3-isobutyl-1-methylxanthine (IBMX), troglitazone (TGZ), Oil Red O solution, and gallic acid were purchased from Sigma-Aldrich (St. Louis, MO, USA). Ellagic acid, chebulagic acid, chebulic acid, and 6-gingerol were purchased from Biopurify (Chengdu, China). Phellopterin was obtained from Assoc. Prof. Dr. Chavi Yenjai, Faculty of Sciences, Khon Kaen University, Thailand. Dulbecco’s modified Eagle’s medium (DMEM with high glucose, L-glutamine, and sodium pyruvate) and fetal bovine serum (FBS) were from Hyclone (Logan, UT, USA). 3-(4,5-dimethylthiazol-2-yl)-2,5-diphenyltetrazolium bromide (MTT), penicillin-streptomycin, and 0.25% trypsin-EDTA were from Gibco (Waltham, MA, USA). Human insulin was obtained from Santa Cruz Biotechnology (San Francisco, CA, USA). The TNF-α was purchased from Abcam (Waltham, MA, USA). The Trizol reagent was from Invitrogen (Waltham, MA, USA).

### 2.2. Plant Materials

**Figure 1:**
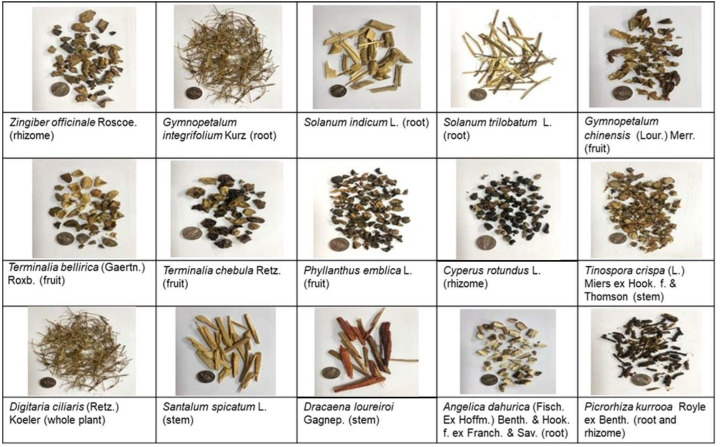
Crude drug of the TPDM6315 recipe.

All 15 plants used in this study are shown in Figure 1. *Gymnopetalum integrifolium*, *Solanum indicum*, and *Solanum trilobatum* were collected from an open field in Srisaket province, Thailand. The remaining 12 were purchased from a traditional herbal drugstore in Khon Kaen province, Thailand. All the crude drugs were authenticated by Assoc. Prof. Dr. Somsak Nualkaew, Faculty of Pharmacy, Khon Kaen University, Thailand. *Santalum spicatum* L. was identified using a TLC chromatogram [28]. The crude drugs were washed and dried in a hot air oven (50 °C). They were kept in a cold room (4 °C) until use. The voucher specimens were deposited in the Faculty of Pharmaceutical Science, Khon Kaen University, Khon Kaen, Thailand.

### 2.3. Preparation of the Extracts

The ethanolic extract (EE) and aqueous extract (AE) were prepared as described by Sabuhom et al. [29]. Each herb constituted 5.26% of the recipe, except *G. chinense*, which constituted 26.32%. The brief procedures are described as follows.

#### 2.3.1. Ethanolic Extract

The dried herbs were ground separately; each herbal powder was mixed and macerated with 95% EtOH in a ratio of herbs: EtOH (1:5 *w*/*v*) three times, with each time lasting for three days. After filtration, the filtrate was concentrated and dried using a rotary evaporator, followed by freeze drying to obtain the EE. It was kept at −20 °C until use.

#### 2.3.2. Aqueous Extract

The dried herbs were cut into small sizes. Then, they were mixed with deionized water in a ratio of herbs: H_2_O (1:25 *w*/*v*) and boiled until one third of the starting volume remained. After that, the mixture was filtered and centrifuged at 7000 rpm at 25 °C for 7 min. The supernatant was freeze dried to obtain the AE. It was kept at −20 °C until use.

### 2.4. HPLC Chromatogram

The HPLC chromatogram was established using an Agilent InfinityLab LC Series 1220 Infinity II LC System, and the peak was detected using a UV detector at a wavelength of 254 nm, as described by Sabuhom et al. [29]. The RP-18 column (Synergi C-18, 250 × 4.6 mm, 4 µm, Phenomenex, Torrance, CA, USA) was used. The mobile phase consisted of solvent A: 0.05% TFA in acetonitrile; gradient with solvent B: 0.05% TFA in water, as follows: 0–10 min: 90%B; 10–20 min: 82%B; 20–30 min: 80%B; 30–35 min: 73%B; 35–40 min: 70%B; 40–45 min: 60%B; 45–50 min: 50%B; 50–60 min: 0%B; and 60–70 min: 100%B. The flow rate was 0.8 mL/min. The peaks were identified by comparing the retention time with the gallic acid, ellagic acid, chebulinic acid, chebulagic acid, phellopterin, and 6-gingerol reference standards.

### 2.5. Evaluation of Anti-Inflammatory Activities of the Extracts in RAW264.7 Macrophages

The RAW 264.7 macrophages were supplied by Assist. Prof. Dr. Pramote Mahakunakorn, Faculty of Pharmaceutical Sciences, Khon Kaen University, Thailand. They were cultured in complete media containing DMEM supplemented with 10% FBS and 1% penicillin-streptomycin in a 5% CO_2_ incubator at 37 °C.

The cytotoxicity of the extracts was determined using an MTT assay [30]. The cells (1 × 10^4^ cells/well) were treated with the extracts in 96-well plates for 24 h. After that, the medium was discarded and 0.5 mg/mL of MTT 100 µL was added and incubated for 2 h. The intracellular formazan crystals were dissolved with dimethyl sulfoxide (DMSO) and the absorbance at 570 nm was measured using a microplate reader (Ensight, PerkinElmer, Waltham, MA, USA). The % cell viability was calculated as follows: % cell viability = (A_sample_/A_untreated control_) × 100, where A_sample_ was the A_570_ of the sample and A_untreated control_ was the A_570_ of the cell incubated with the complete media. The reactions were performed in triplicate. The concentrations of the extracts that provided less than an 80% cell viability were considered as cytotoxic.

Non-cytotoxic concentrations of the extracts were used to investigate the nitric oxide (NO) inhibitory activity. The cells (1 × 10^4^ cells/well) were seeded in 96-well plates for 24 h, then they were treated with the extract in the presence of 500 ng/mL of LPS, and were further incubated for 24 h. After that, the media was assayed for NO using Griess reagent and measured for absorbance at 540 nm. The NO content was calculated from the standard graph of NaNO_2_, y = 0.0079x − 0.0461, R^2^ = 0.995. The percentage of the NO inhibition was calculated using the following formula: % NO inhibition = ((NO_LPS_-NO_sample_)/NO_LPS_) × 100, when NO_LPS_ was the nitrite amount from the LPS group and NO_sample_ was the nitrite amount from the sample treatment in the presence of LPS. The positive control was 250 µM of L-NAME.

### 2.6. The Effects of the Extracts on 3T3-L1 Adipocytes

#### 2.6.1. Cell Culture and Differentiation of 3T3-L1 Adipocytes

3T3-L1 preadipocytes (CL-173, ATCC) were cultured in DMEM completed medium, which was DMEM supplemented with 10% FBS and 1% penicillin-streptomycin, where they were incubated in a CO_2_ incubator at 37 °C, 5%CO_2_. After reaching 100% confluence for 2 days, cell differentiation was performed by changing the medium into DMEM completed medium supplemented with 1 mM of dexamethasone, 0.5 mM of IBMX, and 10 µg/mL of insulin for 2 days, followed by DMEM complete medium with 10 µg/mL of insulin for 2 days, where they were finally incubated in the complete DMEM again for 8–10 days, changing medium every 2 days. The adipocytes were identified by the appearance of triglyceride droplets in the cytoplasm. The cell viability assay using the MTT method was performed on the differentiated adipocytes to obtain the non-cytotoxic concentration ranges of the extracts.

#### 2.6.2. Determination of the Effect of Extracts on Lipid Accumulation in 3T3-L1 Adipocytes

3T3-L1 preadipocytes (1 × 10^4^ cells/well) were seeded in 24-well plates. The treatment with the extracts was performed during the differentiation process. As the preadipocytes were differentiated into adipocytes, triglyceride droplets appeared in the cells. To stain the oil droplets, the cells were washed twice with PBS and fixed with 10% formaldehyde at room temperature for 30 min, then the cells were washed, stained with 0.25 mL of Oil Red O solution, and photographed using an inverted microscope (Nikon). After that, the stained oil was dissolved in DMSO and measured for absorbance at 540 nm using a microplate reader.

The % reduction in lipid accumulation = ((A_diff_ − A_sample_)/A_diff_) × 100, when the A_diff_ was the absorbance of the adipocytes without the sample treatment during the differentiation process and A_sample_ was the absorbance of the sample-treated adipocytes during differentiation.

#### 2.6.3. Effect of the Extracts on TNF-α-Induced 3T3-L1 Adipocytes

3T3-L1 preadipocytes (2 × 10^4^ cells/well) were seeded in 24-well plates and differentiated into adipocytes as described. The differentiated cells were incubated with 10 ng/mL of TNF-α for 24 h to induce inflammation. After that, the extracts and 10 ng/mL of TNF-α were added and the cells were incubated for 48 h. The positive controls were 20 µM of TGZ and 100 nM of insulin.

The glucose content in the medium was determined using a Glucose (GO) assay kit (Sigma-Aldrich, USA) according to the manufacturer’s protocol and calculated from the standard graph of glucose, y = 0.0044x + 0.058, R^2^ = 0.9954.

### 2.7. qPCR Analysis in LPS-Induced RAW264.7 Macrophages and TNF-α-Induced 3T3-L1 Adipocytes

The RAW264.7 macrophages (4 × 10^5^ cells/plate) were cultured in 6 cm cell culture plates for 24 h. After that, they were treated with either the extracts or 500 µM of L-NAME in the presence of 500 ng/mL of LPS for 24 h. The cells were harvested and extracted for their total RNA using Trizol reagent, according to the manufacturer’s protocol.

3T3-L1 preadipocytes (4 × 10^4^ cells/plate) were seeded in 6 cm cell culture plates. After their differentiation into adipocytes, the cells were induced with 10 ng/mL of TNF-α for 24 h. Then, the extracts and 10 ng/mL of TNF-α were added and incubated for 24 h. The cells were harvested and extracted for their total RNA using Trizol reagent. The gene expressions of adiponectin, PPAR-γ, GLUT1, and GLUT4 were evaluated against the treatment group of TNF-α without the extracts. The positive control was 20 µM of TGZ.

The total RNA concentration was measured using a UV spectrophotometer (Biodrop, UK). The first-strand cDNA synthesis reaction of 10 µL from the total RNA 1 µg was performed using a 2-step RT-PCR kit (Vivantis, Malaysia). The relative gene expression was achieved using a real-time PCR machine (CFX 96 Optics Module, Bio-Rad, Singapore). The 10 µL reaction was prepared using Maxima SYBR Green qPCR Master Mix (Thermo Scientific, Lithuania), which contained 2x master mix 5 µL, 10-fold diluted cDNA 3 µL, 10 µM of forward primer 0.2 µL, 10 µM of reverse primer 0.2 µL, and nuclease-free water 1.6 µL. The PCR cycle was 95 °C for 3 min, followed by 40 cycles of 95 °C for 20 s, 55 °C for 20 s, and 72 °C for 30 s. The β-actin gene was used as a housekeeping gene. The relative gene expression was calculated using the 2^−ΔΔCT^ method [31]. The primers used in this study are shown in Table 2.

### 2.8. Statistical Analysis

Statistical significance was determined using GraphPad Prism 8.0.2 (GraphPad Software, La Jolla, CA, USA). A one-way analysis of variance was used to determine the significant differences between the control and treatment groups. The significant differences among each group were detected using a two-way analysis of variance. The post hoc test was performed using Turkey’s test. *p*-values of <0.05 and <0.01 were considered as significant.

## 3. Results

### 3.1. Characterization of the Recipe Extracts

**Figure 2:**
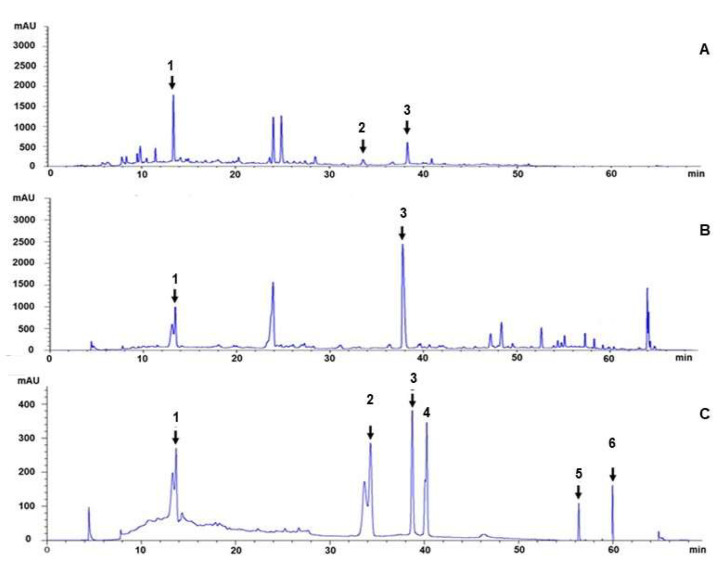
HPLC chromatograms of TPDM6315 extracts. (**A**): AE; (**B**): EE; and (**C**): a mixture of standard substances (peak 1: gallic acid; peak 2: chebulagic acid; peak 3: ellagic acid; peak 4: chebulinic acid; peak 5: 6-gingerol; and peak 6: phellopterin).

An HPLC chromatogram of the ethanolic and aqueous extracts (EE and AE) indicated that the main compounds were gallic acid and ellagic acid (Figure 2). The peaks from EE chromatogram showed those of non-polar substances region, while they were absence in AE. Gallic acid was found to be predominant in the AE, while ellagic acid was the predominant compound in the EE. However, there were several unidentified peaks in the HPLC chromatogram, which play a role in the bioactivity of the formulation.

### 3.2. Anti-Inflammatory Effects on RAW264.7 Macrophages

#### 3.2.1. NO Inhibitory Activity of TPDM6315 Extracts

**Figure 3:**
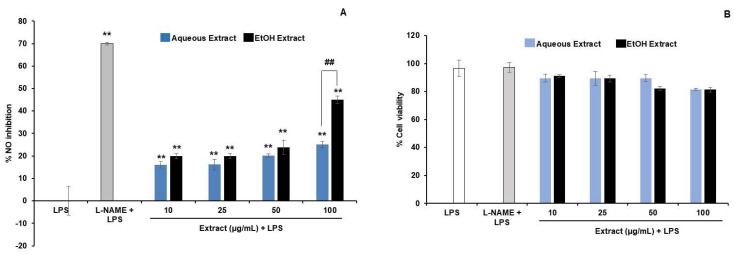
Effect of TPDM6315 extracts on LPS-induced-RAW264.7 macrophages. (**A**): Inhibition of nitric oxide production (the percentage of nitric oxide reduction from the LPS group). ** *p* < 0.01 compared to the LPS group, ^##^ *p* < 0.01: showed the significantly different effect between aqueous extract (AE) and ethanolic extract (EE), *n* = 3. (**B**): Cell viability of LPS-induced-macrophage RAW264.7, compared to the LPS group. No significant difference appeared. Data are presented as mean ± SD. The positive control 250 µM L-NAME was used.

The AE and EE were found to be non-cytotoxic in the concentration range of 10–100 µg/mL in the presence of LPS, as the % cell viability was higher than 80 (Figure 3B). At 10–50 µg/mL, both extracts showed a comparable effect on the NO reduction, while at 100 µg/mL, the EE was significantly more potent than the AE (Figure 3A).

At concentrations of 50 and 100 µg/mL, both the AE and EE exhibited high effects in a dose-dependent manner. Therefore, these concentrations were used for the investigation of the gene expression.

#### 3.2.2. Effect of TPDM6315 Extracts on Gene Expression in LPS-Induced RAW264.7 Macrophages

**Figure 4:**
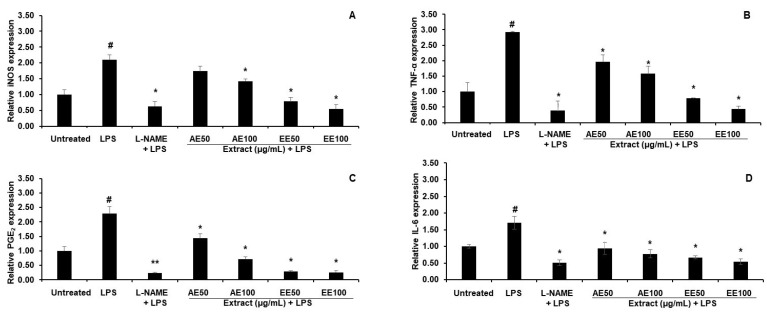
qPCR analysis of gene expression levels from the effects of TPDM6315 extracts on LPS-Induced RAW264.7 macrophages. ((**A**): iNOS; (**B**): IL-6; (**C**): PGE_2_; and (**D**): TNF-α. Data are presented as mean ± SD, ^#^ *p* < 0.05 compared to the untreated group, * *p* < 0.05 compared to the LPS-induced group using 500 ng/mL LPS for 24 h; *n* = 3), 250 µM L-NAME was used as a positive control. ** *p* < 0.01.

LPS stimulated the gene expressions of iNOS, TNF-α, PGE_2_, and IL-6 in comparison to the untreated RAW264.7 macrophages. The AE and EE, at concentrations of both 50 and 100 µg/mL, downregulated these genes, with the EE providing a larger effect, as shown in Figure 4. The reductions in the expressions of the genes related to inflammation and fever pathways supported the anti-inflammatory and antipyretic actions of TPDM6315.

### 3.3. Reduction of Lipid Accumulation in 3T3-L1-Adipocytes

**Figure 5:**
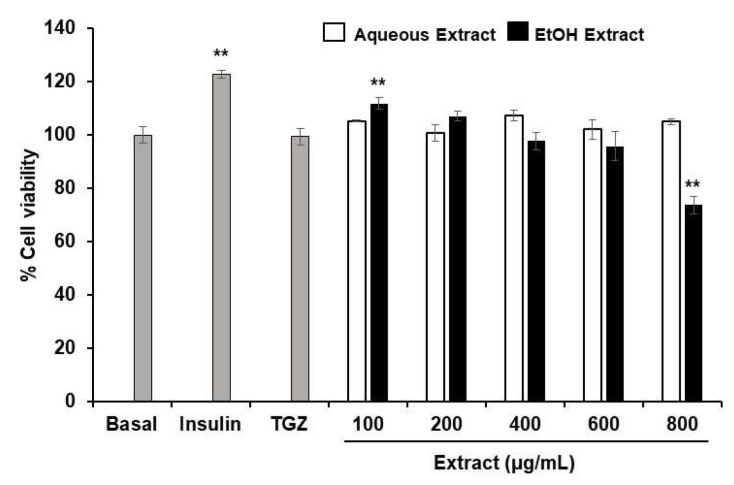
Effects of AE and EE on the percentage cell viability of 3T3-L1 adipocytes after incubation for 48 h. Data are presented as mean ± SD; ** *p* < 0.01 compared to basal group, *n* = 3; 20 µM TGZ and 100 nM insulin were used as positive controls.

**Figure 6:**
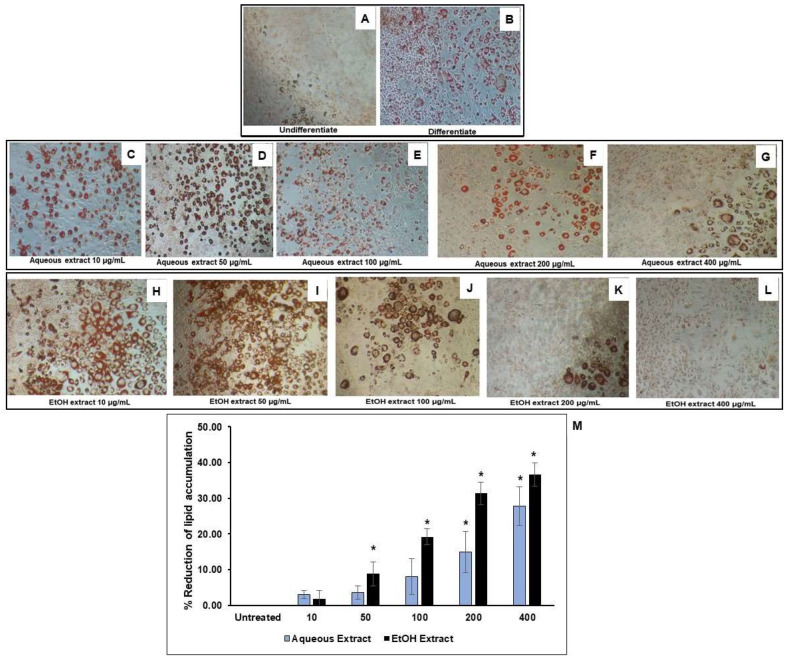
Effect of TPDM6315 extracts, AE and EE, on lipid accumulation in 3T3-L1 adipocytes. The oil droplets were stained with Oil Red O and photographed using an inverted microscope (×10 magnification), ((**A**): 3T3-L1 preadipocyte; (**B**): untreated 3T3-L1 adipocyte; (**C**–**G**): treatment of AE (10, 50, 100, 200, and 400 µg/mL, respectively) during cell differentiation; (**H**–**L**): treatment of EE (10, 50, 100, 200, and 400 µg/mL, respectively) during cell differentiation; and (**M**): % reduction in intracellular oil with AE and EE in 3T3-L1 adipocyte compared to the untreated 3T3-L1 adipocyte; * *p* < 0.05 compared to the untreated 3T3-L1 adipocyte; *n* = 3).

TPDM6315 is composed of several herbs that have been reported to have anti-obesity properties; therefore, the effect of this recipe’s extracts on the intracellular lipid storage in adipocytes was investigated. The non-cytotoxic concentrations of the AE and EE were found to be up to 800 and 600 µg/mL, respectively, as shown in Figure 5. The differentiation of the 3T3-L1 preadipocytes led to adipocytes containing oil droplets of triglyceride, which could be observed microscopically using Oil Red O staining. The addition of the AE and EE during the differentiation of the preadipocytes to adipocytes reduced the lipid accumulation in the adipocytes in a dose-dependent manner, by 3–28% for the AE and by 9–37% for the EE, compared to the untreated differentiated adipocytes, as shown in Figure 6. Significant reductions in the lipid storage were seen at 200–400 µg/mL for the AE and 50–400 µg/mL for the EE.

These results indicated that the AE and EE possessed potential anti-obesity activities, and these activities were more pronounced in the ethanolic extract.

### 3.4. Effects of TPDM6315 Extracts on TNF-α-Induced 3T3-L1 Adipocytes

#### 3.4.1. Effect on Gene Expression 

**Figure 7:**
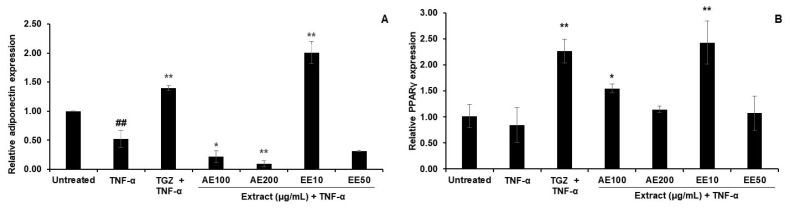
The effect of AE and EE on relative gene expression in TNF-α-induced 3T3-L1 adipocytes. (**A**): adiponectin; and (**B**): PPAR-γ; 10 ng/mL of TNF-α was used to induce inflammation, and 20 µM troglitazone (TGZ) was a positive control. Data are presented as mean ± SD; ^##^ *p* < 0.01 compared to the untreated group; * *p* < 0.05 and ** *p* < 0.01 compared to the TNF-α group; *n* = 3.

Inflammatory mediators such as TNF-α cause the inflammation of adipocytes. The results showed that the TNF-α significantly downregulated adiponectin and decreased PPAR-γ expression (Figure 7A,B), which indicated the inflammation of the adipocytes. Troglitazone (TGZ), a PPAR-γ agonist, augmented the mRNA expression levels of both genes in this model. Of the treatments, only that with 10 µg/mL of the EE showed an enhanced expression of adiponectin, while 10 µg/mL of the EE and 100 µg/mL of the AE both increased the PPAR-γ expression (Figure 7A,B). This indicated that the EE and AE had anti-inflammatory properties in the inflamed adipocytes.

#### 3.4.2. Effect of TPDM6315 Extracts on Glucose Uptake in TNF-α-Induced 3T3-L1 Adipocytes

The inflammation of adipocytes can progress to other consequences of metabolic syndrome such as insulin resistance. Therefore, the glucose uptake ability and related gene expression levels were determined in the TNF-α-induced 3T3-L1 adipocytes.

**Figure 8:**
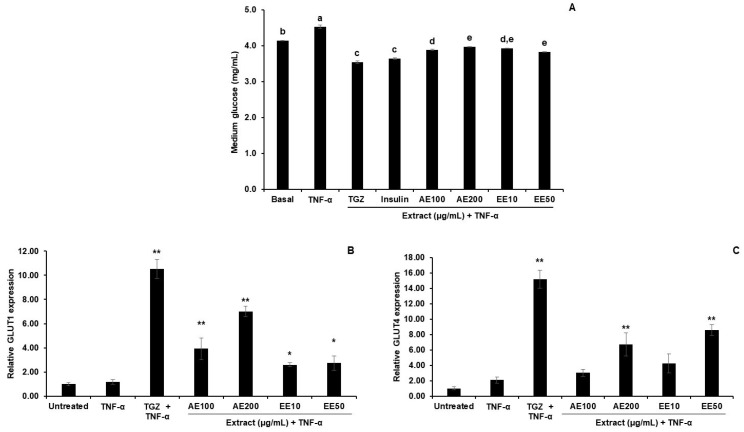
Effect of TPDM6315 on glucose uptake in TNF-α-induced 3T3-L1 adipocytes. (**A**): Glucose content remained in the cell culture media. (The dissimilar letter represented a significant difference between groups (*p* < 0.05), *n* = 3); (**B**): relative GLUT1 expression; and (**C**): relative GLUT4 expression. TNF-α 10 ng/mL was used to induce inflammation, and 20 µM troglitazone (TGZ) was a positive control. Data are presented as mean ± SD, * *p* < 0.05, ** *p* < 0.01, compared to the TNF-α group; *n* = 3. (Glucose medium was as follows: basal: 4.14; TNF-α: 4.5; TGZ: 3.5; insulin 3.6; AE100: 3.9; AE200; 4.0; EE10: 3.9; and EE50: 3.8 mg/mL).

The reduction in the glucose amount in the culture medium indicated glucose uptake into the cells. The higher glucose content in the medium of the TNF-α untreated group than that in the basal group demonstrated that TNF-α suppressed this glucose uptake, as shown in Figure 8A. This might result from the inflammation of the adipocytes progressing to insulin-resistant conditions. The AE and EE both enhanced the glucose uptake of the TNF-α-induced 3T3-L1 adipocytes, as shown by the significantly lower glucose content in the medium of the treated cells compared to that of the TNF-α group. These findings reveal that the AE and EE exerted a recovery effect on the TNF-α inflamed adipocytes in the same order as TGZ and insulin.

TNF-α produced non-significant changes in the glucose transport gene type 1 and 4 (GLUT1 and GLUT4) expression levels, while the PPAR-γ agonist, TGZ, dramatically increased the expressions of these genes. The AE and EE effected an increase in the GLUT1 and GLUT4 mRNA levels. The EE was a more potent stimulator of GLUT1 and GLUT4, as it provided a more significant effect at lower concentrations than the AE (Figure 8B,C). The effects of increasing these GLUT1 and GLUT4 expression levels were consistent with the recovery of the glucose uptake by TPDM6315 in the TNF-α-induced adipocytes.

These results indicated that the AE and EE increased the expression of the glucose transport proteins (GLUTs) rather than affecting the anti-inflammatory effects in the TNF-α-induced 3T3-L1 adipocytes. The lower glucose content remaining in the culture media in both the AE and EE groups demonstrated that the AE and EE attenuated the effect of TNF-α by enhancing the glucose uptake via GLUT1 and GLUT4 (Figure 7A). This might have resulted from the anti-insulin resistance properties of the AE and EE. Further experiments should be performed to clarify the underlying mechanisms of this.

## 4. Discussion

TPDM6315 is a Thai traditional medicine (TTM). It is used as an anti-fever drug and prepared via boiling in water until one third of the volume of the extract is obtained. In this study, an AE and EE were prepared by using different forms of plant raw materials. The AE was prepared using the conventional method of this recipe, which used small pieces of herbs, while the EE was prepared using a maceration of powdered drugs in ethanol. Both extracts consisted of substances ranging from higher polarities in the AE to lower polarities in the EE. The biological activities of the AE and EE demonstrated the overall effects of TPDM6315. However, the extract that showed more efficacy will be further developed into dosage forms. Several herbal constituents in this formula have shown in vitro and in vivo anti-inflammation and anti-obesity effects, as shown in Table 1, leading to the potential ability to prevent or treat the consequences of metabolic syndrome originating from obesity.

Thai traditional recipes generally contain several herbal constituents. When considering the biological activities of an individual herb, new drug indications of the recipe could be hypothesized, which have never been recorded in the textbook. For example, Prasaplai, a Thai traditional medicine consisting of 10 herbs, is traditionally used to regulate menstrual flow. It was found that most herbs in Prasaplai exhibit anti-inflammatory effects, especially Plai (*Zingiber cassumunar*), and Prasaplai extracts possess COX inhibitory activity [32]. These have led to clinical studies proving a new indication of anti-dysmenorrhea [33], which has been successfully established.

In this study, besides supporting the use of the TPDM6315 recipe for anti-fever and determining its potential anti-obesity effects by investigating the anti-inflammatory activity in its macrophages and reducing the lipid accumulation in its adipocytes, it was interesting to explore the anti-inflammatory effects of TPDM6315 on TNF-α-induced adipocytes, in order to evaluate the possibility of a novel use of this recipe for the treatment of metabolic diseases such as insulin resistance.

Although decoction is the conventional method for preparing this recipe for oral administration, the method of maceration with ethanol yielded a more polar range of compounds. Hence, the bioactivity assays were performed on both the aqueous and ethanolic extracts to cover all the polarity ranges of the chemical constituents in this recipe.

### 4.1. Characterization of the Extracts

The biomarkers used to characterize the TPDM6315 extracts in this study possess anti-inflammatory, anti-obesity, and improved insulin-resistant properties. These were gallic acid [34,35,36], chebulagic acid [37,38], ellagic acid [39,40,41], chebulinic acid [42,43,44], phellopterin [45], and 6-gingerol [46,47]. The HPLC chromatogram showed the principal peaks of the gallic acid and ellagic acid, while the peaks of the chebulagic acid, chebulinic acid, phellopterin, and 6-gingerol appeared as minor peaks and needed to be further confirmed. The major compound in the AE was gallic acid, and the EE consisted mainly of ellagic acid.

Gallic acid, ellagic acid, chebulinic acid, and chebulagic acid are phenolic acids in *T. chebula*, *T. bellirica*, and *P. emblica*. Phellopterin is a furanocoumarin of *A. dahurica* root [45] and 6-gingerol is a pungent compound from *Zingiber officinale* [46]. There were also unidentified compounds that could play a role in the bioactivity of the formulation, such as fulgidic acid from *C. rotundus* [9], etc.

### 4.2. Anti-Inflammation Effect of TPDM6315 Extracts

Inflammation is one of the mechanisms of fever. The exogenous pyrogen lipopolysaccharide (LPS) stimulates fever and activates macrophages to produce various pro-inflammatory mediators and pyrogenic cytokines, such as NO, IL-1β, IL-6, TNF-α, PGE_2_, and COX-2 [48,49]. Chronic mild inflammation of adipocytes can lead to insulin resistance, which can progress to diabetes [50].

In this study, a reduction in NO was shown as the final effect of the iNOS function. This reduction in NO suggested the anti-inflammatory effect of the tested sample, which might have come from enzyme inhibition or the reduction in the iNOS protein. The increased gene expressions of PGE_2_, iNOS, IL-6, and TNF-α in the LPS-stimulated RAW264.7 macrophages was in line with previous studies [51,52], in that LPS elevates gene and protein expression, which could be decreased by treatment with anti-inflammatory substances. PGE_2_ is synthesized in response to fever induced by LPS and is the target for antipyretic treatment [53]. iNOS is an enzyme that produces NO in response to inflammation and infection. NO is involved in the modulation of thermoregulation and fever. TNF-α is the first pyrogenic cytokine that occurs after LPS induction and is critical for the fever response of animals and humans. IL-6 is required for fever production and might be involved in fever maintenance [54]. The AE and EE reduced the expressions of PGE_2_, iNOS, IL-6, and TNF-α, which presented the potential anti-fever abilities of TPDM6315 (Figure 4). Several herbal ingredients of TPDM6315 possess anti-inflammatory properties, such as *T. bellirica* [20], *T. chebula* [22], *T. crispa* [24], *Z. officinale* [25], and *P. kurroa* [15]. Furthermore, the major biomarkers of TPDM6315, gallic acid and ellagic acid, are anti-inflammatory substances that inhibit LPS-induced NO, PGE_2_, and IL-6 at both the gene and protein levels [55]. Therefore, in this study, it could be primarily proposed that the recipe extracts might provide anti-inflammatory effects. Further experiments to detect the protein and cytokine levels are needed to prove this mechanism.

Several traditional Thai recipes used to relieve fever have been reported for their anti-inflammatory actions. For example, Ya Ha Rak’s ethanolic extract inhibits the NO production in LPS-induced RAW264.7 macrophages with an IC_50_ 40.4 µg/mL [56], and Prasachandaeng’s ethanolic extract suppresses the TNF-α production in LPS-activated RAW264.7 [57]. Furthermore, Juntaleela has been shown to reduce inflammation in rat ear swelling and carrageenan-induced rat paw models [58]. Hence, our results might provide some evidence to support the use of the TPDM6315 formula to treat fever.

### 4.3. Effect of AE and EE on Lipid Accumulation of 3T3-L1 Adipocyte

Many herbs in the TPDM6315 recipe have anti-obesity properties. Obesity causes chronic low-grade systemic inflammation that releases TNF-α and leads to insulin resistance, which further enhances adipogenesis and lipid accumulation [59].

The 3T3-L1 murine preadipocyte is commonly used to evaluate adipogenesis. Adipogenesis is characterized by the occurrence of the intracellular accumulation of lipid droplets [60]. A differentiation of the 3T3-L1 preadipocyte can be induced by using a mixture of IBMX, FBS, and dexamethasone. However, the spontaneous adipogenesis of preadipocytes without induction can also be observed [61]. Fat accumulation results from adipogenesis and an imbalance between lipogenesis and lipolysis. The process of adipogenesis involves the expression of key adipogenic transcription factors, such as peroxisome proliferator-activated receptor gamma (PPAR-γ) and CCAAT/enhancer-binding protein alpha (C/EBPβ). Anti-obesity effects can primarily be evaluated through a reduction in the amount of triglyceride oil droplets in adipocytes. This reduction can come from an inhibition of adipogenesis, a decrease in lipogenesis, or via the acceleration of lipolysis. The treatment with the AE and EE reduced the fat accumulation in the adipocytes, indicating possible anti-obesity properties for this recipe, and this effect was correlated with the activities of several herbal constituents of TPDM6315 as shown in Table 1 that can reduce fat accumulation in adipocytes. Further experiments should be conducted to investigate this mechanism of action, including a determination of the glycerol release and triglyceride content to indicate lipolysis, as well as a measurement of the expression of the genes related to adipogenesis and lipogenesis.

Therapeutic agents that inhibit adipogenesis could potentially prevent and treat obesity. For example, the aqueous extract of the Thai traditional recipe, Jatupalathika, has been shown to inhibit fat accumulation and adipogenesis and induce lipolysis in 3T3-L1 adipocytes [62]. Three of the four herbs in Jatupalathika (*T. chebula*, *T. bellirica*, and *P. emblica*) are also included in TPDM6315. Therefore, these herbs might play essential roles in the possible anti-obesity effects of TPDM6315. On the other hand, the predominant active compounds in the TPDM6315 recipe have potential anti-obesity properties. For example, ellagic acid inhibits adipogenesis by suppressing the terminal differentiation and lipid accumulation in 3T3-L1 adipocytes [63]; gallic acid increases insulin sensitivity and reduces obesity [36]; chebulinic acid acts as a potent antiadipogenic agent suppressing the differentiation of 3T3-L1 preadipocytes into mature adipocytes [43]; and 6-gingerol inhibits adipogenesis in 3T3-L1 cells [64].

### 4.4. Effect of AE and EE on TNF-α-Induced 3T3-L1 Adipocytes

The correlation between obesity, adipose tissue inflammation, and metabolic diseases makes anti-inflammatory properties an interesting target for treating obesity-related metabolic complications [65]. For example, well-known anti-inflammatory drugs, such as aspirin, can improve insulin sensitivity [66].

The chronic inflammation of adipose tissue induces insulin resistance, which suppresses PPAR-γ expression and impairs adipogenesis. The results from Figure 6 showed that TNF-α decreased the adiponectin and PPAR-γ expression levels in the 3T3-L1 adipocytes, which were in the same direction as previous studies [67]. TNF-α, when added to differentiated adipocytes, causes a downregulation in the PPAR-γ mRNA expression [68] but does not alter the mRNA stability [69].

Adiponectin is secreted from adipocytes and acts as an anti-inflammatory agent and insulin sensitizer [70]. The increase in the adiponectin expression of the adipocytes following the treatment with 10 µg/mL of the EE might indicate an anti-inflammatory effect of the extract against TNF-α-induced inflammation and a protective effect against the development of inflammatory responses [23].

PPAR-γ is a transcription factor expressed predominantly in adipose tissue that plays a crucial role in adipogenesis. Troglitazone, a PPAR-γ agonist, exerts its anti-inflammatory effects by suppressing NF-kB and is also able to improve insulin sensitivity [71]. In the present study, an upregulation of PPAR-γ was observed with the treatment with the TPDM6315 extracts (AE100 and EE10) and TGZ. These findings revealed a potential mechanism for TPDM6315, with a possible function of the PPAR-γ agonist that could be used to treat insulin resistance in type 2 diabetes [72].

The reduction in glucose uptake and downregulation of adiponectin in the TNF-α-induced 3T3-L1 adipocytes might be associated with insulin resistance. TNF-α inhibits glucose uptake and GLUT4 protein expression [73]. The highest amount of glucose content in the TNF-α-induced 3T3-L1 adipocytes in this study (Figure 8A) suggested a possible insulin-resistant condition and could be recovered via treatment with TGZ, insulin, EE, and AE.

The TNF-α did not significantly decrease the GLUT4 and GLUT1 mRNA expressions in comparison to the basal group. This might be due to the short incubation period (24 h). However, a suppression of the glucose uptake was shown by the higher glucose content in the medium of the TNF-α-induced adipocytes in comparison to that of the basal group (untreated adipocytes). It has previously been reported that prolonged exposure (72–96 h) to TNF-α results in more than an 80% reduction in GLUT4 mRNA [4]. Further experiments on insulin resistance and the effects of TPDM6315 should be performed.

## 5. Conclusions

TPDM6315 reduced the nitric oxide production and expressions of the COX_2_, PGE_2_ TNF-α, and IL-6 genes, indicating a possible anti-inflammatory effect of this recipe, which has been traditionally used to treat fever. The biological activities of several herbs in this recipe indicated a potential ability for TPDM6315 to prevent obesity, which was supported by a decreased lipid accumulation in TPDM6315-treated adipocytes. Furthermore, the anti-inflammatory effects of TPDM6315 on adipocytes stimulated with TNF-α and the induction of GLUT1 and GLUT4 expressions might prevent a progression to insulin resistance. More experiments are needed to be performed to explain these findings.

## Figures and Tables

**Table 1:** The herbal constituents of the TPDM6315 polyherbal formula and their reported anti-inflammation, anti-pyretic, and anti-obesity effects.

Botanical Name	Part	Traditional Use as Anti-Fever *	Anti-Inflammation and Antipyretic	Anti-Obesity
*Angelica dahurica* (Fisch. Ex Hoffm.) Benth. & Hook. f. ex Franch. & Sav.	Root	/	[7]	[8]
*Cyperus rotundus* L.	Rhizome	-	[9]	[10,11]
*Digitaria ciliaris* (Retz.) Koeler	Whole plant	/	-	-
*Dracaena loureiroi* Gagnep.	Stem	/	[12]	-
*Gymnopetalum chinense* (Lour.) Merr.	Whole fruit	/	-	-
*Gymnopetalum integrifolium* Kurz	Root	/	-	-
*Phyllanthus emblica* L	Whole fruit	-	[13]	[14]
*Picrorhiza kurrooa* Royle ex Benth.	Root and rhizome	/	[15]	[16]
*Santalum spicatum* L.	Stem	/	-	-
*Solanum indicum* L.	Root	/	[17]	[18]
*Solanum trilobatum* L.	Root	/	-	[19]
*Terminalia bellirica* (Gaertn.) Roxb.	Whole fruit	/	[20]	[21]
*Terminalia chebula* Retz.	Whole fruit	/	[22]	[23]
*Tinospora crispa* (L.) Miers ex Hook. f. & Thomson	Stem	/	[24]	-
*Zingiber officinale* Roscoe.	Rhizome	/	[25]	[26]

* data from Thai traditional medicine textbook [27].

**Table 2:** Primers used in this study.

Gene	Forward Primer	Reverse Primer
iNOS (112 bp)	5′ CTGCCAGGGTCACAACTTTAC 3′	5′ AACAGCTCAGTCCCTTCACC 3′
PGE2 (143 bp)	5′ TGACAGCCGTGGGTAAAGAC 3′	5′ CCAAGGCTGGATGTGTGAGT 3′
TNFα (128 bp)	5′ GATCGGTCCCCAAAGGGATG 3′	5′ TTGCTACGACGTGGGCTAC 3′
IL6 (154 bp)	5′ GTCCTTCCTACCCCAATTTCCA 3′	5′ TAACGCACTAGGTTTGCCGA 3′
Adiponectin (146 bp)	5′ TGACGACACCAAAAGGGCTC 3′	5′ ACCTGCACAAGTTCCCTTGG 3′
GLUT1 (139 bp)	5′ CAATGGCGGCGGTCCTATAA 3′	5′ TGTAACTATGCGTCTCCCGC 3′
GLUT4 (200 bp)	5′ TCTGACGTAAGGATGGGGAAC 3′	5′ TTGTGGGATGGAATCCGGTC 3′
β-Actin (196 bp)	5′ GACACGAGTTGGTTGGAGCA 3′	5′ GCGACCATCCTCCTCTTAGG 3′

## Data Availability

Not applicable.
